# Deciding about the use of a Personal Safety Alerting Device—The need for a legitimation process: A qualitative study

**DOI:** 10.1111/jan.14566

**Published:** 2020-10-13

**Authors:** Friederike J. S. Thilo, Jos M. G. A. Schols, Ruud J. G. Halfens, Monika Linhart, Sabine Hahn

**Affiliations:** ^1^ Applied Research & Development in Nursing Department of Health Professions Bern University of Applied Sciences Bern Switzerland; ^2^ School CAPHRI Department of Health Services Research Maastricht University Maastricht The Netherlands; ^3^ School CAPHRI Department of Family Medicine Maastricht University Maastricht The Netherlands

**Keywords:** ageing in place, community‐dwelling, decision‐making, empowerment, focus group, gerontechnology, independent living, nurses, patient safety, technology adoption

## Abstract

**Aims:**

To explore reasons, thoughts, motives, and influencing factors regarding the use or non‐use of Personal Safety Alerting Devices (PSADs) in the daily lives of community‐dwelling older persons.

**Design:**

A qualitative descriptive study design was used.

**Methods:**

Six focus groups were conducted with a total of 32 older persons between February–August 2016. Data analysis followed the Qualitative Analysis Guide of Leuven.

**Results:**

The participants described the use or non‐use of PSADs as a decision resulting from a “legitimation process”. This process implies that a person needs to perceive the necessity for a PSAD and then determine the right moment at which to start using it. During this process, each person weighs her or his “ageing self” and “perception of technology” then decides whether to start using a device or to delay its use. “Critical events” initiate this process, compelling the person to consider their own safety and their possible need for assistance.

**Conclusion:**

The legitimation process suggests that the initiation of PSAD use represents a turning point in life. Using a PSAD is not simply a matter of obtaining one. It is a complex decision‐making process establishing legitimation for its use, which is interwoven with one's individual ageing, self‐perception, and the meaning attributed to the device.

**Impact:**

Older persons need to be supported; in particular, they require time to go through the legitimation process for PSAD use. Nurses can empower them in this process, such that they perceive using a PSAD as a means to restore their frailty balance and feel enabled to (re)gain control over their own life and thus to preserve their independence.

## 摘要

**目标:**

探讨社区老年人在日常生活中使用或不使用个人安全警报装置(PSAD)的原因、想法、动机和影响因素。

**设计:**

采用定性描述性研究设计。

**方法:**

2016年2月至8月,对共计32名老年人参加的六个核心小组进行调查。遵循鲁汶的定性分析指南对数据进行分析。

**结果:**

参与者对使用或不使用个人安全警报装置的说明作为“合法化过程”的决定。这个过程意味着,一个人需要意识到个人安全警报装置的必要性,然后决定何时开始使用。在这个过程中,每个人都会对其“自我衰老感”和“技术认知”进行权衡,然后决定是开始使用该设备还是推迟使用。“关键事件”会启动这一过程,迫使该人员考虑自己的安全问题和可能的援助需求。

**结论:**

合法化过程表明,开始使用个人安全警报装置代表着一个生命的转折点。使用个人安全警报装置并不仅仅是获得个人安全警报装置这么一回事。这是一个复杂的决策过程,为其使用确立合法性,与个人的衰老感、自我认知以及设备的意义紧密关联。

**影响:**

老年人需要得到支持;特别是,他们需要时间通过个人安全警报装置使用的合法化程序。护士可以在这一过程中获得赋权,便于他们可以将使用个人安全警报装置作为恢复脆弱平衡的一种手段,并感到能够(重新)掌控生活,保持独立性。

## 1 INTRODUCTION

“Ageing in place” is embraced by health care and political agendas worldwide (International Federation on Ageing, 2011). Ageing in place promotes the well‐being of older persons by enabling them to live independently, while safely and socially integrated in their accustomed surroundings in the community (Scharlach & Diaze Moore, 2016).

Community care is often considered a precondition for ageing in place (Vasunilashorn et al., 2012) and community nurses specifically facilitate and support ageing in place (Greenfield et al., 2019). Community nurses assist older persons in maintaining their activities of daily living, in identifying any early symptomatic changes and in enabling a safe way of life (Smolowitz et al., 2015; Young et al., 2015).

## 2 BACKGROUND

The safety of older persons is of primary concern for community nurses, as many falls occur in the home environment (Hefny et al., 2016; National Council for Aging Care, 2018). International studies show that the rate of falls is between 25%–35% for community‐dwelling older persons older than 65 years of age. This age group experiences at least one fall per year (Gillespie et al., 2012) and fall rates are up to twice as high for persons aged 75 years and older (Gale et al., 2016). A fall is “an unexpected event in which the participants come to rest on the ground, floor, or lower level” (Lamb et al., 2005). A considerable number of older persons, from 53%–80%, are unable to get up after a fall and 13–30% of them endure a so‐called “long lie” (Fleming et al., 2008; Simpson et al., 2014), i.e. lie on the ground/floor for longer than one hour, leading to a high risk for adverse outcomes, such as hypothermia, hospital admission, or serious injuries like hip fractures or head trauma (Bloch, 2015; Gill et al., 2013).

Therefore, Personal Safety Alerting Devices (PSADs) are pivotal for safe ageing in place. PSADs can reduce health‐threatening consequences by enabling rapid assistance in emergency situations (Agboola et al., 2017; Nyman & Victor, 2014). Even though older persons consider PSADs to be helpful or report being satisfied with them, they are still rarely used in the daily life (Heinbüchner et al., 2010; McLean, 2016; Nyman & Victor, 2014). Non‐usage, minimal‐usage, or refusal of usage has been shown to be related to usability problems such as difficulty in activating an alert, economic issues, or forgetting to wear/activate the device (Heinbüchner et al., 2010; Stokke, 2016).

Although increasing international attention is being given to the “need‐driven” development of PSADs by involving older persons (Chaudhuri et al., 2015; Thilo et al., 2016, 2018), the discrepancy between their acceptance and their non‐usage/minimal usage, continues to be a significant healthcare challenge (Lapierre et al., 2018; Nyman & Victor, 2014; Stokke, 2016). Several researchers have reported that the acceptance of technology by older persons can be influenced by health professionals (Peek et al., 2014; Stokke, 2016). For instance, community nurses’ perceptions of the usefulness and appropriateness of assistive technologies for patient care was shown to be meaningful to older persons (Piscotty et al., 2015). However, there is a gap in the current literature regarding the understanding of the influencing factors around PSAD use and non‐use in daily life. Far too little attention has been given to the reasons and motives of community‐dwelling older persons. Gaining more insight may generate new strategies as to how community nurses can support older persons in PSAD use, thus enhancing safe ageing in place.

## 3 THE STUDY

### 3.1 Aim

The aim of this research was to answer the question: *What are the reasons, thoughts, motives and influencing factors regarding the everyday use and non‐use of a PSAD from the perspective of community‐dwelling older persons?*

### 3.2 Design

A qualitative descriptive research design was adopted (Kim et al., 2017; Sandelowski, 2010), using focus groups (Stewart & Shamdasani, 2015) to explore and uncover factors and rationales for behaviour related to PSAD use and non‐use. Most research focused on the acceptability and usability of technologies is quantitative in nature and conducted in disciplines other than health care and nursing; thus, the context of acceptable technologies for older persons has hardly been investigated (Holden & Karsh, 2010; Marangunić & Granić, 2015; Taherdoost, 2018). Furthermore, a qualitative approach sampling the perspective of the user group itself was chosen as most likely to advance understanding.

### 3.3 Sample/participants

Through purposeful and snowball sampling, community‐dwelling older persons who were ambulatory, German‐speaking, and aged 75 or older were recruited. The age limit was based on data indicating that adults 75 years of age and older are part of a late technology adopters generation (Smith, 2014) and also because they are at a high risk for falls (Rubenstein, 2006). Moreover, the participants should have had some experience with PSADs or with situations of falls or fear of falling. Persons using a wheelchair, living in an institution or an assisted‐living facility, or who were cognitively impaired were excluded.

It is well‐known that recruiting community‐dwelling older persons for study participation is challenging (Hawranik & Pangman, 2002). We contacted over 25 organizations such as seniors’ associations or healthcare service organizations by distributing printed and electronic information leaflets. Faculty members, who were not part of the research team or in other research project collaborations, were contacted via email and asked to forward the leaflet to their (grand‐) parents, other relatives, and neighbours. Additionally, older persons who had already participated in a previous study from the Institute of Nursing Science were contacted. We assumed that they might be interested in participating in a new study due to the thematic proximity of falling.

### 3.4 Data collection

We organized a focus group once at least four participants agreed to participate in the study. Six focus groups with four to eight participants each were conducted between February–August 2016. Each discussion lasted 2 hr and was audio recorded. All focus groups were moderated by the first author (FJST), who is experienced in qualitative interviewing and were assisted by a research assistant. Different strategies were used to foster group dynamics and interactions: emphasizing that personal and conflicting viewpoints were welcomed, active listening, follow‐up questions, or non‐verbal signs (eye‐contact, nodding) (Brinkmann & Kvale, 2015).

A topic‐guide was used. The sequence of a focus group discussion is displayed in Table 1.

**TABLE 1:** Sequence of a focus group discussion

Focus group		
	First part:	Topics addressed: ▪ Everyday experiences with electronics ▪ Experiences with the topic of fall/PSADs ▪ One's own view of being a user or non‐user of a PSAD
	Presentation of ten PSADs ▪ An emergency button; ▪ A watch; ▪ A house emergency call combined with an alert bracelet or necklace; ▪ A mobile phone with speed dial buttons; ▪ A senior‐friendly telephone combined with an alert bracelet or necklace; ▪ A sensor mat for a chair and for the floor; ▪ A radio transmitter fall detector; ▪ An infrared sensor; ▪ A camera‐based‐system; ▪ A wearable fall detection sensor (prototype)	
	Second part:	Topics addressed: ▪ Reasons for liking or disliking a PSAD ▪ Reasons for considering a device as helpful/pleasant or awkward/unpleasant ▪ Any additional thoughts regarding PSAD use and non‐use
	Short questionnaire on socio‐demographics and technology use	

The presentation of ten PSADs (Table 1) stimulated the discussion, provided information on PSAD diversity, use and function, allowed hands‐on experiences and better understanding of reasons for liking or disliking a PSAD or considering it helpful or awkward. Finally, study sample participants completed a short questionnaire.

Following each discussion, field notes were made, and a research diary was kept (FJST) to document the study process and decisions made. The moderator and research assistant reflected on the themes discussed. Insights were incorporated into subsequent focus groups.

### 3.5 Ethical considerations

The study was conducted in accordance with the Swiss Federal Act on Research Involving Humans, confirmed by the cantonal Ethics Committee in October 2015. Written and verbal informed consent were obtained prior to study participation. The study leaflet explained the purpose of extending knowledge on falls and currently available personal alerting technologies. This study was conducted in compliance with the protocol, the current version of the Declaration of Helsinki, the ICH‐GCP or ISO EN 14155 (as far as applicable), as well as with national legal and regulatory requirements.

### 3.6 Data analysis

Each focus group discussion was transcribed verbatim, using the software program f4^®^. Identifying information was pseudonymized. Data were analysed using the Qualitative Analysis Guide of Leuven (QUAGOL), which allows a comprehensive and systematic but flexible analysis process consisting of two parts: in‐depth preparation of the coding process and the actual coding process (Dierckx de Casterlé et al., 2012). The QUAGOL was supplemented in steps seven to nine, using the methods of open and axial coding and memo writing (Boehm, 1994; Saldana, 2016). Please refer to Table 2. This combination allowed for enhanced depth of interpretation during data analysis.

**TABLE 2:** Data analysis and interpretation process

Methods		Focus group (FG) *n* = 6	Researchers
QUAGOL Steps 1–5 carried out independently: thorough (re)reading; narrative report; from narrative report to inductive developed conceptual topic scheme; fitting‐test of the topic scheme; constant comparison process, summary list of themes consolidated by mutual consensus		FG 1 to 6 FG 1–3–5 FG 2–4–6	FJST; BH; CG
QUAGOL Step 6: Summary list of themes ‐> discussion of inductively developed themes and consolidation by mutual consensus		FG 1 to 6	FJST, SH; ML
QUAGOL Steps 7–8: coding process using list of themes (back to the data); analysis of themes (meaning, dimensions & characteristics) and memo writing; Supplemented by: open coding (questions to the text, verification of additional/other inductive themes), and axial coding (matrix coding family comprising conditions, context, consequences and strategies to identify “the” phenomenon), consolidation by mutual consensus	Iteratively		
QUAGOL step 9: extraction of the essential structure Supplemented by: axial coding (as described above)			
QUAGOL step 10: description of the findings			FJST; SH; ML; JMGAS; RJGH

The first author (FJST) was responsible for data analysis. Steps seven to nine (Table 2) occurred iteratively until the phenomenon was identified and a nuanced understanding of the themes grounded in the data was achieved. These steps were mainly carried out by the first author and continuously and critically discussed and reflected on with two senior researchers (SH, ML) experienced in qualitative research. Step ten of the QUAGOL was critically discussed and reflected on in the research team. Table 3 shows a data trail of the themes developed according to the QUAGOL process. From step seven data management was supported by MAXQDA software (VERBI GmbH, Berlin, Germany; Version 12 and 2018).

**TABLE 3:** Exemplarily data trail of themes developed according to the QUAGOL process

Method QUAGOL			
See also description of Table 2		Examples (not comprehensive)	
Preparation of Coding Process	Steps 1–5: narrative report of each FG, based upon inductively developed summary list of themes per FG	FG 2: choosing the right moment; influence of health professional and relatives; deciding oneself FG 5: safety need; social network; being allowed to die; being reserved about technology FG 6: perception of technology based on experience; positive and negative influence of relatives; criteria of the “right moment”	
Actual Coding Process	Step 6: Consolidated list of themes of all six FGs	FG 1–6: “the” right moment; health status; safety need; relational status; technology experience; technology attitude; recognizing fall/risk of fall as a problem; device as “stigma of ageing”; the challenge of ageing; being allowed to die	
	Steps 7–8 (iteratively with step 9): coding process using the list of themes from step 6 (FGs 1–6)	Health status FG1/3−321 Participant (P) 5: I would say frailty. Several Ps: mhm agreeing. P3: well‐being, health condition P1: losing one's mind P2: yes, yes P1: suddenly, you have trouble knowing where you are. Several Ps: mhm agreeing. P4: dementia or something like that P1: Alzheimer, yes P4: In those cases, it (PSAD) would be very reasonable, for sure. FG5/360 P2: I think there are days when you feel better, you can take a lot. Then there are days when you feel dizzy or unwell. Then you think: now you must do something. It depends on your [physical] form for the day. Then there are days when it goes great again, then I don't even think about something like that (PSAD).	
		Safety need FG2/1,095−1,098 P2: So, somewhere the stamp is cancelled (image for: life is finite). Then you must go. I think we can't insure everything in our life, that's unfortunately the case. P3: No, no. P2: There is no hundred percent security either. FG6/359−362 P1: Well, I’m going to say something nasty. P4: Go ahead. P1: Until you fall «on your face» for the first time. And then maybe your rethinking will come. Then, you say, I will feel safer when I have such a thing (PSAD) [so that] help comes quickly. It is very difficult to think oneself into another situation when someone is physically still very well off. P4: Yes, it is individual.	
		The challenge of ageing FG4/504 P3: Life is uncertain. P1: Yes, Yes. P3: You can't do everything… P1: Yes, Yes. P3: If you're just scared, it's not good. Interviewer (I): Mhm P1: Then he (a friend) said: watch out, don't think too much about that stuff (devices) otherwise you'll get the feeling that now, you need that (PSAD) too. Several Ps: Mhm, yes. I: that goes in the direction of that you prefer not to deal with it and you think nothing will happen to me? Several Ps: Laughing, yes, yes. P1: Well, but he said there are those who take it so seriously (…). P3: A lot of things are simply very abstract, even growing old, suddenly you're 80 years old, but it's not quite so tangible. It seems to me that you just think a little too little about it. FG3/388−393 P3: I have already experienced it myself, so my husband and I are still quite active. But then he had a back operation once and then the other one is looking out. But if the other one is also not well off at the same time, it will be bad. Then you notice how quickly you reach your limits. (...) It isn't a straight‐line hike, what do you do then? Mostly it goes up again sometime, but the older you get, the more such situations happen. And when you have had something serious, then it doesn't get quite as good before anymore, it always leaves some effect. P1: Yeah, you can't get back on your feet as before. P3: Yes, exactly! P1: You just stay a little bit behind of what has been before. P2: yes, that's it! P1: And it can suddenly go very fast.	
		Technology attitude FG3/244 Everything that is so ‘highly technical’, I already have a kind of defense, an inner defense, yes. FG5/91−97 P4: I would like to say that my husband came in the beginning (of the ‘computer age’), he is probably the oldest here, and when he stopped working, he said that a computer would never come into our house. He'd had it up to here. And I stuck to it for about eight years. Until I just felt like, no way, I don't want to be left behind. I: Mhm P4: I have fun and am interest in technical things. I: Yes P4: And, also considering that the family was not nearby, I had no help from them, I just got into (these technical things).	
	Step 9 (iteratively with steps 7–8): extraction of the essential structure (FGs 1–6)	Figure 1: Legitimation process “The need to perceive its necessity”; Critical Events for Use and Non‐use; Ageing Self; Perception of Technology; Decision	
	Step 10: description of the essential findings	See section results	

### 3.7 Rigour

Scientific rigor was ensured through a variety of techniques (Engward & Davis, 2015; Lincoln & Guba, 1985) such as memo writing, using a coding paradigm (see QUAGOL steps 6–8) and keeping a reflexive record of the decisions made during analysis. Weekly discussions of the analysis process (FJST, SH, ML) ensured that the emerging findings were credible, that the findings were grounded in the data and that a critical stance in the analysis and interpretation was maintained. Credibility and confirmability were enhanced by means of researcher triangulation (FJST, BH, CG) in the first six steps and by on‐going debriefing throughout the entire process.

## 4 FINDINGS

### 4.1 Participant characteristics

In total 32 community‐dwelling persons, including 24 women, aged 75–90 (mean 82) participated in the study. Eight participants had a history of falls in the prior 12 months and 12 participants sometimes experienced fear of falling. One participant was currently using a PSAD. Further characteristics are displayed in Table 4.

**TABLE 4:** Participant characteristics (*N* = 32)

Characteristics	Participants (*N* = 31^a^)
Age (years), mean (*SD*; min‐max)	82 (4.25; 75–90)
Abbreviations: *SD*, standard deviation; min, minimum; max, maximum	
	*n* (%)
Gender	
Female	24 (77.4)
Male	7 (22.6)
Overall health	
Rather good	10 (32.3)
Good	20 (64.5)
No answer	1 (3.2)
History of fall(s) in the last 12 months	
Once	6 (19.4)
Twice	1 (3.2)
Three times or more	1 (3.2)
No	22 (71)
No answer	1 (3.2)
Experiencing fear of falling	
Sometimes	12 (38.7)
No	18 (58.1)
No answer	1 (3.2)
Experiencing instability while walking	
Sometimes	15 (48.4)
No	16 (51.6)
Walking aid usage	
No	27 (87.1)
Yes	4 (12.9)
Household situation	
Living alone	15 (48.4)
Living with another person	16 (51.6)
Household size	
Living in a house	7 (21.9)
Living in an apartment	24 (77.4)
Assistance in daily living	
Cleaning	21 (70)
Washing clothes	1 (3.3)
Shopping	1 (3.3)
Transport	4 (13.3)
Medication	2 (6.5)
Usage of electronic devices/ Internet	
Smartphone use	7 (22.6)
iPad/Tablet use	6 (19.4)
Laptop use	8 (25.8)
PC/Computer use	13 (41.9)
Mobile phone use	10 (32.3)
Internet use at home	21 (67.7)

^a^ One participant did not fill in the questionnaire.

### 4.2 Decision process on use versus non‐use of a PSAD in daily life

The analysis identified an iterative decision‐making process comprising an interplay of three core themes: *“Critical Events”*, “*Ageing Self”,* and *“Perception of Technology”*. Each of these themes were then further described using 3–6 subthemes, which were marked in bold and italicized in the text. This interplay illuminated the participants’ reasoning processes before they ultimately decided whether or not to use a PSAD in their daily life.

#### 4.2.1 Critical events

The participants mentioned that *critical events*, often situations related to safety issues in everyday life, caused them to reflect on their need for assistance and on PSAD use and non‐use. Several critical events were highlighted: They reported that *experienced and reported falls* of others, initiated thoughts about their own risk. Some of those falls had no health‐related consequences, while others included severe injuries or were even fatal, as the person was not found immediately. Some participants intended to use a PSAD after a first fall, while others preferred to wait as long as possible:

I postpone it [use of PSAD] and I am very careful not to fall. (FG4/139/P2).

Participants felt that if someone lived with a partner without cognitive impairments, or was in daily contact with another person, they did not need a PSAD, because they were looking out for one another. Living with another person was described as providing a feeling of safety and confidence and that, if required, mutual help would be provided. As soon as a partner was no longer present, they noted, the situation changed (***loss of a close relationship***) and they started to question their safety.

***Concern of relatives*** was another critical event. Participants said that mostly their children suggested the use of a PSAD, because they were worried about the parent's safety. However, the participants emphasized that their children were more fearful about their safety than they themselves were. Thus, several added that they would use a PSAD for the sake of their children, because it represented a kind of reassurance for them. Nevertheless, relatives’ concern might not entirely legitimate its use:

I know a lady, whose children feel permanently stress, because she falls frequently. However, she refused to wear a (safety) watch. Her children gave her one; she put it on for several days and then she threw it away. (FG3/50/P3).

Interestingly, when thinking about who might use a PSAD, the participants consistently differentiated between older persons who were cognitively fit and in good physical health and those who were not (***perceived deterioration of health***). It seems that this distinction is crucial in establishing what would justify the use of a PSAD, for example, being cognitively impaired, or having physical health issues, such as dizziness or frailty. Although participants mentioned this clear criterion of use, most of them underlined that their current physical and cognitive health did not yet require PSAD use. However, self‐perception might contrast with an external view:

Just because I stagger a bit in the street ‐ this is not a reason for such a device, I am too young, it is too early, even if I will turn 78 soon. (FG1/445/P1).

Furthermore, ***perceived deterioration of mobility***, for example, instability or insecurity while walking, was mentioned by some of the participants as another event which would make them, or already made them, reflect on their own safety. Physical limitation like a low level of mobility was considered a suitable reason for using a PSAD.

Most of the focus groups brought up the topic of dying. It was the only critical event explicitly mentioned as a possible reason for non‐use (***longing for death***). They expressed a kind of serenity or fatalism regarding their “remaining” life. Dying was characterized as a natural process of life; however, the discussions remained ambivalent. The participants emphasized, on the one hand, that suffering should be avoided:

If you have a broken leg, it's nicer to be rescued after 2 hr than to lie in pain all day because you can't call anyone. (FG5/431/P1).

On the other hand, some insisted one had to accept one's death and that destiny should decide what will happen in the case of an emergency:

If it is necessary, I must endure pain. I have reached an age where dying is close…I don't want to live 20 more years. […] I have reached the age (for dying), without doubt! That's why, I am not impressed by those things (PSADs). (FG3/294/P1).

#### 4.2.2 Ageing self

The second core theme, the ageing self, was raised and intensively discussed by all participants. Interestingly, discussing PSAD use and non‐use spontaneously evoked the topic of ageing across all focus groups. They emphasized that ageing is unavoidably linked to declining health and physical changes and that these can occur “out of the blue”. As a way of ***dealing with age‐related changes***, some avoided thinking about them, as if doing so might influence their emotions negatively. Additionally, not thinking about age‐related changes might help to prevent them. Several participants were, however, convinced that anticipating ageing would enable them to cope more appropriately and that it might help them with living independently (e.g., with the use of a PSAD).

Although knowing that one should ideally use a PSAD before something happened, most participants admitted thinking:

Well, basically you would need it [PSAD] before it happens [fall or emergency]. But, you think: I still have time. (FG6/303/P3).

This moment of PSAD use is ideally situated in the future as it is linked to the moment of requiring assistance, which is attached to the transition from a person “growing older” to one “being old” (***becoming a person requiring assistance***):

Well, I guess that the feeling at the back of one's mind is: as soon as I use a [PSAD] device, I perceive myself as already old. (FG4/156/P4).

The transition towards becoming a person requiring assistance seems to evoke changes in self‐perception and challenge the person to re‐define themselves (***re‐defining the self***). Using a PSAD was discussed as transitioning from being independent to becoming dependent. It seems to be a considerable step to admit to oneself that one requires assistance and is old:

I knew a lady, she was already 80 years old and she always travelled with a “normal” train ticket. I said to her: why aren't you buying a (cheaper) train ticket for seniors? She replied: well, the train conductor doesn't need to know that I am that old! (FG1/865/P2).

#### 4.2.3 Perception of technology

Technology yielded the third core theme. Our analysis revealed that the process of deciding whether or not to use a PSAD can be influenced by an individual's perception of technology. Many of the participants described themselves and their generation as being strangers in the world of today's technologies. Consequently, they felt disconnected and sometimes socially excluded, particularly when they read phrases like “further information is available online”. Some participants reported feeling overwhelmed and experiencing fear and negative emotions, as in:

I don't want to admit that I have no clue about […] the new technologies anymore. Because I was never a fool (laughing) and now I realize that the others are better than me and I must admit that I’m a fool in the context of new technologies. (FG1/813/P5).

Participants who used digital devices frequently, however, experienced it as being life‐facilitating, especially when communicating with their children and grandchildren.

I often text with my grandchildren. And now we have a son who is in Asia for four years. I’ve also learned to skype. (FG6/87/P5).

Their ***experiences*** were closely linked to their ***attitudes*** towards new technology. Several participants felt favourable towards technology, since it could make life more interesting. Others described themselves as having negative attitudes towards technology. For example, they depicted themselves as being suspicious, in opposition to or being against technology.

Furthermore, ***device characteristics*** were key to their perception of technology. Most of the participants emphasized that a PSAD should be nice to look at and feel. Wearing a device on the body is highly attractive, as it is easily “accessible” for use and can be hidden. A panoply of devices should be on offer and should take into consideration age‐related changes such as impaired sight, hearing and motor skills, as well as reduced reaction time and learning speed. Additionally, the range of reliable alert transmission should include both indoors and outdoors, to maintain mobility. The most crucial device characteristic, however, was its ease of use. This was summarized as being able to manipulate a PSAD without thinking, as falling induces stress, which often hinders clear thinking.

Finally, the alerting process and the person who comes to give assistance should be clearly defined; health professionals were explicitly preferred as contact persons, as they are available 24 hr a day and trained for emergencies. The feeling of safety can be supported by personal voice contact when an alert is triggered. Another important PSAD‐related characteristic was how and where to find an overview of available PSADs, along with related “neutral” information about their strengths and limitations (e.g. tested by health professionals). Most participants were unsure as to where to access PSADs and obtain information. They would prefer to touch, test, play with, and receive advice about the most suitable model for their individual needs and requirements (e.g. relatives, neighbours, or housing). Becoming familiar with the available devices in a non‐binding way would support the decision‐making process.

### 4.3 PSAD use and non‐use—a decision process requiring legitimation

Based on our data analysis, a decision process regarding PSAD use and non‐use in daily life was identified that comprised the interplay of the three core themes: *Critical Events*, *Ageing Self,* and *Perception of Technology*. Furthermore, the study revealed that this decision process required an additional aspect which was called *“legitimation”*. Participants needed to perceive the necessity of using a PSAD, as illustrated in the following statement:

It is clear for me. As soon as [a PSAD] is necessary for me and as soon as I get the feeling that, yes, something might happen and nobody would notice me, well, I would buy such a watch. But, still, I really don't perceive its necessity. (FG3/83/P2).

**FIGURE 1:**
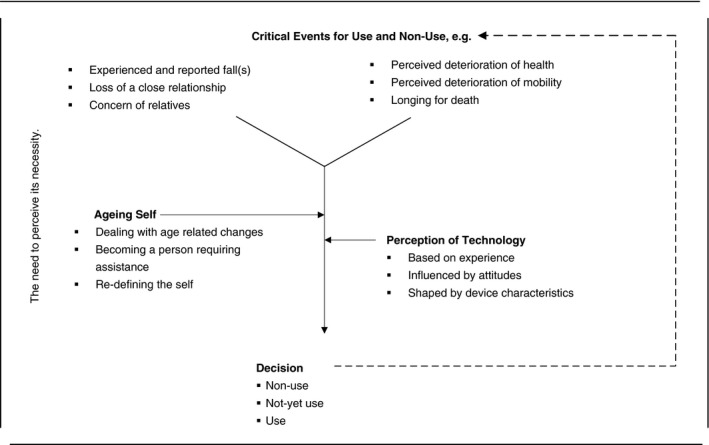
Descriptive model of the legitimation process of the use or non‐use of a PSAD

Thus, this decision process can be termed a *“Legitimation Process”*, which is initiated by a critical event compelling the older person to reflect on their perceived safety, on their need for assistance and the right moment to start using a PSAD in daily life. During the legitimation process the older person weighs up their *‘Ageing Self’* and their *‘Perception of Technology’* as summarized in Figure 1.

## 5 DISCUSSION

This study focused on the reasons, thoughts, motives, and factors influencing the use and non‐use of PSADs in daily life from the perspective of community‐dwelling older persons. The findings revealed that the decision to use or not use a PSAD is the result of a legitimation process which involves an interplay of the ageing self, e.g. dealing with age‐related changes and the person's perception of technology, e.g. their technology experience. The legitimation process is initiated by a critical event, which causes the person to reflect on her/his own safety and a possible need for assistance. This process leads the older person to a decision about PSAD use or non‐use. As time goes on, depending on new critical events, another cycle of the legitimation process may be re‐initiated.

The process was labelled a legitimation process, as the use of a PSAD requires reasons to support its perceived necessity. As long as this necessity is not perceived by the older person, s/he will decide on “not‐yet use” or non‐use. It can be argued that the device is not yet appropriate, unlike for others who are in poorer health or in unsafe living situations. This finding concurs with the literature: older persons, in general, appreciate technology, but are convinced that current users are older persons and that they are not yet “that old” (Holender et al., 2018). It seems that participants implicitly postponed the initial usage of a PSAD, so that they could transition as late as possible into this group of “really frail and dependent” older persons. This observation has important implications for nursing practice. Older persons want to stay in control and thus influence the decision of PSAD acceptance or rejection. Nurses need to involve older persons in the decision‐making, provide PSADs information about (dis‐)advantages and usage, adapted to the individual living context and accord time for the decision.

The findings suggest that a critical event where the individual experienced feelings of insecurity and/or helplessness is required to initiate the legitimation process for using a PSAD. A fall is often followed by fear of falling, which may in turn lead to a need for support (De San Miguel et al., 2015; Trotman & Morriss‐Roberts, 2016). This finding suggests that nurses should systematically assess critical events, to evaluate the possibility for initiation of PSAD usage.

Surprisingly, the concern expressed by relatives could be described as an ambiguous critical event. Relatives might facilitate PSAD use, but this study revealed that such concern may lead to ambivalence among older persons. Some decide to put relatives’ minds at ease by starting to use a device, which is in line with the findings of Stokke (2016). However, our study also revealed that relatives’ concern is not automatically related to regular PSAD use and can even turn into non‐use. Recent research suggests that children, being driven by worry, try to convince their parents to purchase and use technology, which can make older persons feel coerced (Luijkx et al., 2015). Therefore, it could be argued that although relatives tend to promote PSAD use, they can also be a reason for its non‐use. This contradiction might be explained by the legitimation process elucidated by our study: an older person needs to personally legitimise PSAD use and perceive its necessity before deciding to own one. Thus, the study extends current knowledge by suggesting that although relatives may play a crucial role in the acquisition of a PSAD, they might also be a reason for its non‐use. Further research should shed more light on this promoting and hindering influence of relatives.

The topic of longing for death emerged, somewhat curiously, in the discussion on PSAD use and non‐use. It is hardly unexpected that people in their eighties ruminate on dying. What is surprising is that long lies and increased morbidity after a fall seem to be blanked out and replaced by the notion that “a fall is fatal”. This finding could be interpreted as an indication that older persons lack knowledge about long lies and their consequences, as well as about the function of a PSAD in this situation. Prior research reveals that older persons have difficulty accessing PSADs (Stokke, 2016) and thus might have idiosyncratic or partly incorrect assumptions regarding PSADs. This finding implies that nurses should provide appropriate information regarding falls, fall consequences, and what the added value of a PSAD can be, enabling their older clients to take an informed decision on whether to use a PSAD.

Another pivotal finding was the strong need to perceive the necessity of using a PSAD, before deciding to use it. This is consistent with previous research on assistive technologies (Chen & Chan, 2011; De San Miguel et al., 2015; Peek et al., 2014). Since technology acceptance in older persons remains a challenge (Schulz et al., 2015), it seems important to ask what perceived necessity might signify from the perspective of PSAD users themselves. The ageing self was a dominant topic in the focus groups; therefore, the findings suggest that PSAD use and ageing are closely related. The literature describes the process of ageing as partly dealing with the maintenance of a sense of self, which can be affected by apparently ‘superficial’ events, such as giving up a driver's license or other long‐standing activities (Lloyd et al., 2014). As striving for independence and maintaining control over one's life is central to older persons’ selfhood (Hale et al., 2010), it is conceivable that a PSAD evokes similar losses in the self‐concept of older persons. Consequently, the core influencing factor concerning PSAD use does not seem to be technology, but, instead the process of ageing and the older person's perception and attitude towards ageing. Additionally, participants clearly differentiated between older persons who are cognitively and physically fit and those who are not. This implies that those who are not fit are the ones who should use a PSAD. It could be further deduced that the participants equated PSAD use with “becoming‐a‐dependent‐person”, substantiating previous findings, where persons in their eighties described the use of a walker, cane, or wheelchair as “crossing a boundary into old age” (Heikkinen, 2000). These key insights imply that a PSAD cannot be considered a “simple” gadget in the everyday life of community‐dwelling older persons.

### 5.1 Limitations

Some limitations should be considered when interpreting the findings. One might be the purposeful and snowball sampling method. However, data collection and analysis supported data saturation, which equates with rich and thick data (Fusch & Ness, 2015). Themes were confirmed across all focus groups. Additionally, conducting six focus groups is considered sufficient to generate adequate and saturated data (Jayasekara, 2012), which can be confirmed by the research team. Since community‐dwelling older persons are difficult to recruit, no strategy to maximise the sample variation within a focus group was used.

PSADs are particularly useful for fall incidents, as rapid assistance can prevent or shorten long lies. However, PSADs can also be used in other emergency situations, such as acute pain or discomfort, or in cases of threat. When interpreting the findings, it should be considered that the participants were introduced to example of fall incidents, but the PSADs presented were also deployable for other emergency situations. Moreover, it is possible that Swiss attitudes to topics like ageing, technology and dying might differ from those in other European, Asian or North American countries.

## 6 CONCLUSION

The study suggests that the initial use of a PSAD represents a turning point in life. This turning point is activated by a critical event, such as a fall, by concerned relatives or other persons and by declining health or decreased mobility. Using a PSAD in everyday life is not simply a matter of obtaining a device. It is a complex process entailing the perception of necessity, which is interwoven with notions of individual ageing, self‐perceptions, and the meanings attributed to the device. Our description of the legitimation process provides an in‐depth understanding of a long and iterative process that allows older persons themselves to accept (or reject) the use of a PSAD. Knowledge about the legitimation process can be used, e.g., in the communication with older persons, to help reflect on their thoughts, fears and questions regarding PSADs. In addition, this knowledge can be applied to develop targeted interventions aimed at enabling older persons to take an informed decision regarding PSAD use.

Additionally, the findings extend current knowledge by revealing that older persons need to be empowered in such a way that they perceive using a PSAD as a means to restore their frailty balance, (re)gain control over their own life and preserve their independence. In this, it is important that nurses clarify the individual advantages of using a PSAD in daily life and explain how and in what situations safe living and perceived independence are supported.

Furthermore, older persons require information regarding the types and functioning of PSADs, as well as where to access them and how to integrate them into daily life. This is important in enabling them to make informed decisions either for or against PSAD use. Using a PSAD has the potential to support ageing in place, despite illness or functional decline. Further research might investigate which interventions are effective in supporting the legitimation process.

## CONFLICT OF INTEREST

No conflict of interests has been declared by the authors.

### Peer Review

The peer review history for this article is available at https://publons.com/publon/10.1111/jan.14566.
